# Estimation of gestating sows’ welfare status based on machine learning methods and behavioral data

**DOI:** 10.1038/s41598-023-46925-z

**Published:** 2023-11-29

**Authors:** Maëva Durand, Christine Largouët, Louis Bonneau de Beaufort, Jean-Yves Dourmad, Charlotte Gaillard

**Affiliations:** 1 PEGASE, INRAE, Institut Agro, 35590 Saint Gilles, France; 2 Institut Agro, Univ Rennes1, CNRS, INRIA, IRISA, 35000 Rennes, France

**Keywords:** Machine learning, Animal behaviour

## Abstract

Estimating the welfare status at an individual level on the farm is a current issue to improve livestock animal monitoring. New technologies showed opportunities to analyze livestock behavior with machine learning and sensors. The aim of the study was to estimate some components of the welfare status of gestating sows based on machine learning methods and behavioral data. The dataset used was a combination of individual and group measures of behavior (activity, social and feeding behaviors). A clustering method was used to estimate the welfare status of 69 sows (housed in four groups) during different periods (sum of 2 days per week) of gestation (between 6 and 10 periods, depending on the group). Three clusters were identified and labelled (scapegoat, gentle and aggressive). Environmental conditions and the sows’ health influenced the proportion of sows in each cluster, contrary to the characteristics of the sow (age, body weight or body condition). The results also confirmed the importance of group behavior on the welfare of each individual. A decision tree was learned and used to classify the sows into the three categories of welfare issued from the clustering step. This classification relied on data obtained from an automatic feeder and automated video analysis, achieving an accuracy rate exceeding 72%. This study showed the potential of an automatic decision support system to categorize welfare based on the behavior of each gestating sow and the group of sows.

## Introduction

Animal welfare may be defined as “the positive mental and physical state linked to the satisfaction of its physiological and behavioral needs, as well as its expectation. This state varies according to the animal’s perception of the situation”^1,2^. In the literature, many researchers have tried to evaluate the welfare of animals on farms. In Europe, one example is the Welfare Quality program, which was developed for the main species of farm animals^3^. However, these protocols, as the animal needs index^4^, the qualitative behavior assessment^5^ and the semantic modelling^6^, evaluated the welfare at group level for feasibility on commercial farm. Few studies have been dedicated to welfare at the individual scale with an evaluation of behavior (social interactions or physical activity level) as a reflection of animals’ emotions^7,8^. Different events may have an impact on animal behavior and physiology, such as in pigs thermal variations^9^, sound emission^10^, enrichment^11^, and feeder competition^12^. These events have an impact on the groups’ behavior. However, variability between animals should be taken more seriously into account, due to the individual perception of an animal confronted with a stimulus^2,7,8^.

Data issued from sensors or automatons used for individual monitoring could be a way to study and automatically evaluate the behavioral response of each animal to an event and its welfare state^13,14^. These technologies are appearing on farms due to the development of precision livestock farming, defined as “the management of livestock production using the principles and technology of process engineering”^15^. The use of sensors or other connected objects (like electronic weight scale or ventilation regulatory system) allows individual monitoring of animals, often based on radio frequency identification (RFID) recognition of the animals^13^. For example, electronic feeders could be used to feed pigs but also to predict disease outbreaks^16^ and tail biting outbreaks^17,18^ at the group level; or to predict body weight at an individual level^19^. To analyze the large amount of real-time data collected by sensors, machine learning methods could be used, involving algorithms learning from data to solve a specific task^20^.

Identifying farm animal welfare is a growing sustainability concern for society that may benefit from automated recording. The aim of this paper was to categorize the individual welfare status of gestating sows based on behavioral data (social behavior and physical activity), including days during which environmental perturbations were induced, employing machine learning techniques. As many welfare studies failed to estimate individual welfare, this study may show a new way to approach the ideal state of really assessing welfare in focusing on the behavioral components of welfare. First, three groups of behaviors (clusters) were defined using a clustering method on the behavioral data. Then, these clusters were interpreted using sow characteristics, health status and environmental conditions to relate to welfare status. Finally, the classification of the gestating sows was performed, using the labeling issued from the clustering, to predict the welfare status of each animal. This classification relied on continuous variables, linked to feeding behavior and postures, extracted from automaton and sensor (feeder and camera) collected during 2 days per week over 6–10 weeks, depending on the group of sows.

## Methods

### General approach

**Figure 1:**
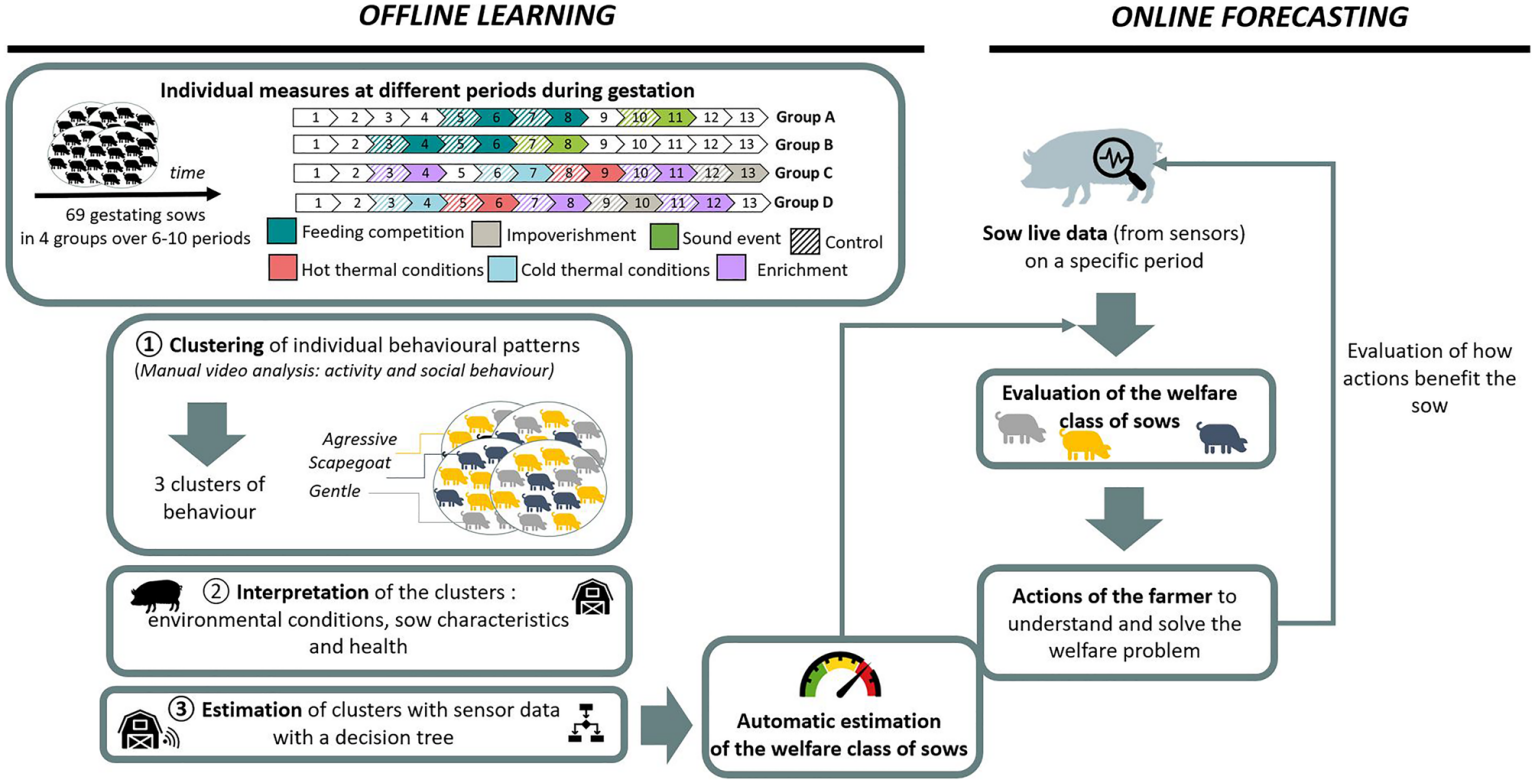
General approach of the current study (offline learning part) and practical perspectives (online forecasting). The number (first box, upper left) corresponds to the week of gestation.

The objective of the study was to estimate the welfare status of each animal on a farm (Fig. 1) in an automatic manner for convenience and cost. Automatons and sensors automatically produce data at an individual scale, linked with the animal’s behavior, which could be used for its welfare estimation. Behavioral data like social behavior^21^, postures and occupation (lying, standing, walking, eating, drinking, exploring behavior) can indeed be used to evaluate some components of the welfare status^22,23^ of an animal or its emotional state^24,25^ and were therefore considered in this study as the true field truth (also called ground truth).

During the offline learning process, a clustering algorithm was applied on behavioral data collected from manual video analysis (Fig. 1). The clustering results suggest grouping the gestating sows with similar behaviors into 3 subgroups (called clusters). This clustering task was performed on a dataset with a total of 388 individual observations from 69 sows, i.e., one sow per period (sum of 2 days per week: Tuesday and Wednesday) on a total of 6 to 10 periods per sows (depending on the group, Fig. 1). These periods corresponded to control periods (i.e., baseline behavior) or event periods with induced perturbations of the environment during a few days (3–5 days) in the week to induce changes in behaviors and welfare status. The idea of these perturbations was to increase the behavior variability between sows.

The method is also composed, during the offline learning process, of the exploration of the behavioral dataset, which aims to produce an interpretable model, i.e., a decision tree. For online forecasting, given some new data on a sow (feeder and automatic video analysis data), the already learned decision tree classifies the sow into one of the welfare groups.

For the offline learning process, the relation of the clustering results with welfare status was interpreted using individual characteristics of the sows (age, body weight, back fat thickness, health status) and the experimental setup (control vs. events). This clustering step enables data annotation, paving the way for the application of a supervised and interpretable machine learning algorithm. Once the data were annotated with the cluster labels, a decision tree was learned on sensor data, feeder and automatic video analysis. The performance of this classification was evaluated using the labels obtained by the clustering step. It's a way of classifying sows into different clusters using other data, i.e. automatically recorded data (feeder and cameras).

For the online forecasting process, the decision tree is used for inference on new live data from the sows (feeder and automatic video analysis data) and provides the predicted welfare class label of the sow. One of the major interests of the decision tree is its interpretability, which makes it a valuable tool for understanding the algorithm decision rules behind predictions.

### Animals and management

This study was carried out from July 2020 to April 2021 at the Pig Physiology and Phenotyping Experimental facility (UE3P, Saint Gilles, France; 10.15454/1.5573932732039927E12) of the French National Research Institute for Agriculture, Food and Environment (INRAE). The experimental protocol was approved by the local Ethics Committee in Animal Experimentation in Rennes (France) and the French Ministry of Higher Education, Research and Innovation (authorization on living animals No. APAFIS 25883-2020070711528084) in accordance with the French legislation on experimental animal care. All methods used on the experimental protocol were carried out in accordance with relevant French and European guidelines and regulations and with ARRIVE guidelines.

A total of 69 crossbred sows (Landrace × Large White), housed in four gestating rooms with one pen inside (groups A, B, C and D), were studied throughout their gestation. The parity of the sows ranged from 1 to 10, included 16 primiparous (4 per group). All sows were confirmed to be pregnant (by ultrasound) 30 days after insemination. After pregnancy confirmation, the number of sows kept per group was between 17 (for group A, B and D) and 18 (for group C). Sows were group-housed from a few days after artificial insemination to almost the end of their gestation at 104 days. The gestating room had a concrete floor enriched with straw and two chains. The space allowance was 3.1 m^2^/sows. Two cameras (RS-CCPOE280IR4-DH, Ro-main Inc., Canada) per room continuously recorded the sows. Ad libitum access to clean water was provided by two electronic drinkers (Asserva, France). Each sow received an individualized ration from two self-locking electronic feeders (Gestal, JYGA Technologies Inc., Canada) able to identify the sows’ RFID ear tag. The quantity of feed supply was calculated individually but fixed for all gestation periods (despite a general increase of 500 g/day from 86 days), while the ration composition was adjusted daily with an individual blend of two diets (a low and a high nutrient content: with a standard ileal digestible lysine of 3.30 and 8.50 g/kg of feed, respectively). Nutrient requirements were calculated using the nutritional model InraPorc^26,27^.

During the gestation period, different events were induced for a few days (starting on Mondays at noon) in the gestation room: a competitive situation for feed (occurring twice), a sound event (occurring once), cold and hot thermal variations (occurring once each), enrichment (occurring twice) and impoverishment (occurring once) of the environment. Each “event week” followed a “control week” (a week without any induced event). The competitive situation for feed was created by closing one of the two available feeders in the gestation room for 5 days and nights^28^. The sound event was induced by the random emission of 40 sounds of 30 s, under 85 decibels, every 10 min, twice a day (from 23:00 h to 04:00 h and from 13:30 h to 18:30 h) for 3 days^29^. These two moments were chosen to correspond to a moment of high activity level (the night, as new feeding day started at midnight) and a moment of low activity level (the afternoon with a resting period). The cold and hot thermal events consisted of setting the thermostatic control at 12 ± 2 °C and 32 ± 2 °C, respectively, using ventilation fans or heaters, for 3 days and nights^30^. The impoverishment was induced by removing the straw bedding on the room. Finally, the enrichments were composed of the addition of straw during the first session and by the supply of jute bags, brush and ropes during the second session.

### Data collection

Every Monday, animal-based measures (number of skin lesions, cleanliness of the sow, and identification of potential health problems) were evaluated based on the Welfare Quality^®^ assessment^3^ with the same trained observer. A sow was categorized as “unhealthy” if, during the measure, a health problem was detected (bursitis, lameness, tail biting, vulvar lesion or abscesses); otherwise, the sow was categorized as “healthy”. The electronic feeders automatically recorded every visit of each sow (time of the day, duration of the visit, feed intake). Extracted data were preprocessed to filter the outliers (i.e., visit duration over 6 h, 0.007% of all the dataset) and aggregated at an individual scale for the 7 variables described in Table 1. The feeder order, i.e. the order in which the sows had access to the feeder, was also recorded and represented a proxy of the hierarchical order^31^ (i.e., rank 1 for the most dominant sows). Manual analysis of videos was carried out in continuous by trained observers to monitor individual behavior (social and activity) for two moments (23:00 h–04:00 h and 13:30 h–18:30 h) of 2 days per period. The detailed ethogram is available in Durand et al.^28^, and raw data are available in a datapaper^32^. From the video analysis, 3 indexes were calculated to obtain a limited number of variables gathering the intensity and the valence of the level of activity and social interactions (Table 1). For example, when the “Index_activity” has a negative value, the sow spent more time passive than active, and when the “Index_giving” has a negative value, the sow gave more negative interactions than positive ones. An automatic analysis of videos was also achieved at a group level using a convolutional neural network algorithm (Dilepix, France)^33^. In that case, data were aggregated on 6 variables as the percentage of sows detected in 6 postures (Table 1).

**Table 1:** Ethogram and description of the features used in the study (*NV* nutritive visit, *NNV* nonnutritive visit, *cat* categories, *Avg* average, *Nb* number).

Features	Description
Features from manual video analysis (used for clustering)	
Active	Total time spent by a sow in active occupation, such as standing, walking, drinking, eating, exploring, or manipulating the enrichment objects
Passive	Total time spent by a sow in passive occupation, such as sitting, lying, or observing
Index_activity	Time spent ‘Active’ minus spent ‘Passive’ divided by the total time spent ‘Active’ and ‘Passive’
Giving positive interactions	Number of times a sow gives positive social interactions, such as huddling (lying with body contact with another sow), achieving “snout to snout” or sniffing
Receiving positive interactions	Number of times a sow receives positive social interactions, such as huddling (lying with body contact with another sow), achieving “snout to snout” or sniffing
Giving agonistic interactions	Number of times a sow gives agonistic social interactions, such as head knocking, pushing, biting, threatening, fleeing or attacking
Receiving agonistic interactions	Number of times a sow receives agonistic social interactions, such as head knocking, pushing, biting, threatening, fleeing or attacking
Index_giving	Number of ‘Giving positive interactions’ minus number of ‘Giving negative interactions’ divided by the total number ‘Giving positive interactions’ and ‘Giving negative interactions’
Index_receiving	Number of ‘Receiving positive interactions’ minus number of ‘Receiving negative interactions’) divided by the total number ‘Receiving positive interactions’ and ‘Receiving negative interactions’
Features from feeder data (used for decision tree)	
Nb_NV	Total number of nutritive visits (with feed consumption) at the feeder per period
Nb_NNV	Total number of nonnutritive visits (without feed consumption) at the feeder per period
Time_NV (in min)	Total duration of nutritive visits at the feeder per period
Time_NNV (in min)	Total duration of nonnutritive visits at the feeder per period
Avg_NNV (in min/visit)	Duration of nonnutritive visits divided by the number of nonnutritive visits
Avg_NV (in min/visit)	Duration of nutritive visits divided by the number of nutritive visits
Rank_cat	Sow order of visit to the feeder (indicator of hierarchical status)
Postures features from automatic video analysis (used for decision tree)	
Side_lying (%)	% of sows in lateral lying position (4 legs on the same side, flank fully on the ground)
Ventral_lying (%)	% of sows in ventral lying position (1 or more legs not visible or on the same side, flank not fully touching the ground)
Standing (%)	% of sows on the 4 legs (still or moving) only on contact with the floor
Sitting (%)	% of sows with the chest off the ground, the front legs straight and the back legs on the ground
Eating (%)	% of sows in the feeder (door closed and head in the trough)
Drinking (%)	% of sows in the drinker (head and two forelegs in the trough)

The behavioral data (from the manual and automatic video analysis and from the feeder), recorded on the same time windows, were aggregated in sums over a period of 2 days (Tuesday, Wednesday) per week to exclude days with animal manipulation.

### Clustering, nonsupervised data mining method

Clustering algorithms group similar data together based on their intrinsic characteristics without any prior knowledge of the class labels. For this study, the unsupervised algorithm used for clustering was K-medoids. Instead of using the mean value as the center of a cluster, K-medoids clustering uses the actual data point that minimizes the total dissimilarity to all other points within the cluster. The medoid is then less affected by noise and outliers (abnormal values). K-medoid clustering helps create more accurate and robust clusters and is a suitable choice when dealing with real-data applications where noise and outliers are a concern^34^. The principle of the clustering algorithm is first to initialize the cluster centers randomly or according to some criteria. Then, the algorithm iteratively assigns data points to one of the clusters based on a similarity or distance measure and updates the cluster centers until convergence. When applying a clustering algorithm, the parametrization requires selecting appropriate values for various parameters that affect the clustering approach. These parameters may include the number of clusters to generate, the distance metric used and the convergence criteria for stopping the iterative process. Choosing optimal values for these parameters is crucial because it impacts the quality of the clustering results^34^.

K-medoid clustering was performed on the dataset composed of three variables extracted from the manual analysis video (‘ratio_activity’, ‘ratio_receiving’, ‘ratio_giving’) and normalized before clustering (values between − 1 and 1). The appropriate number of clusters (from 1 to 10) was 3 for the clustering task, as it gave the highest performances with the following metrics: inertia, silhouette coefficient and graph, elbow graph, Calinski‒Harabasz index and Davies‒Bouldin index^34^. The distance metric used was Euclidean. The algorithm initialized the centroids using the k-means++ method, which resulted in better initialization and potentially improved the clustering performance^35^.

### Decision tree, supervised classification method based on clustering label

The supervised classification method chosen was a decision tree applied to the data labeled by the classes issued from the previous clustering step. A decision tree is a popular machine learning algorithm that learns from input data and uses a tree-like structure to make decisions or predictions. In a decision tree, each internal node represents a combination of feature values, and each branch corresponds to a possible value or outcome. Decision trees are commonly used for classification tasks and provide interpretability and ease of understanding due to their graphical representation^36^. The dataset composed of feeder data and automatic video analysis data was randomly split into a training dataset (70% of the original dataset) and a test dataset (30%), with care taken to ensure that there were a sufficient number of observations for each cluster. The hyperparameter chosen for the maximum depth was 3 (1 to 5) due to their optimal performance on the training dataset^35^. The decision tree was created with the feeder data (7 variables at the individual scale) and automatic video analysis data (6 variables at the group scale).

### Statistical analyses and implementation of algorithms

The effect of factors (week, group, event) on the clusters was evaluated using Cochran’s *Q* test, which was designed for paired data with more than two groups for comparison. Friedman tests and post hoc tests were used to assess the effects of continuous data (body weight, age, backfat thickness) on the clusters. The threshold for statistical significance was set at P < 0.05 and trend-level significance between 0.05 < P < 0.10. The implementation was realized in Python using the scikit-learn library^35^ (version 1.2.1.) for the clustering (K-medoid) and the classification (decision tree) tasks. Statistical analyses were performed using the Python ‘statsmodels’, ‘scipy’ and ‘scikit_posthocs’ libraries.

## Results

### Behavioral patterns identified by clustering

The inertia of 3 clusters was 189.07 (better than with 5 clusters = 157.95), and the silhouette score was 0.33 (better than with 5 clusters at 0.30). The three clusters (0, 1, and 2) gathered 95, 131, and 162 observations of sows per period, respectively (Table 2). The medoid of cluster 0 was characterized by a passive activity level (ratio_activity = − 0.14), a balanced number of given interactions between positive and negative (ratio_giving = 0.08), and more negative interactions received (ratio_receiving = − 0.75). Sows of cluster 0 were therefore called ‘scapegoats’. The medoid of cluster 1 was characterized by a high activity level (ratio_activity = 0.07) and giving and receiving more negative interactions (ratio_giving = − 0.80; ratio_receiving = − 0.67). Sows of cluster 1 were called ‘aggressive’. The medoid of cluster 2 was characterized by a passive activity level (ratio_activity = − 0.18), giving more positive interactions (ratio_giving = 0.5) and receiving a balanced amount of positive and negative interactions (ratio_receiving = 0). Sows of cluster 2 were called ‘gentle’.

**Table 2:** Differences in features between the three clusters. The letters a, b, and c show a significant difference for post hoc tests (*NV* nutritive visit, *NNV* nonnutritive visit, *cat* categories, *Avg* average, *Nb* number).

	Cluster 0 (scapegoat)	Cluster 1 (aggressive)	Cluster 2 (gentle)	All clusters	SEM	P value
Number of observations	95	131	162	388		
Features from manual video analysis (used for clustering)						
Index_activity	− 0.14	0.13	− 0.17	− 0.06	0.02	0.84
Index_giving	0.08^a^	− 0.73^b^	0.51^c^	− 0.02	0.03	< 0.001
Index_receiving	− 0.72^a^	− 0.62^b^	0.04^c^	− 0.37	0.02	< 0.001
Features from feeder data (used for decision tree)						
Nb_NV	2.48^a^	3.11^b^	2.39^a^	2.66	0.05	0.02
Nb_NNV	10.40^a^	15.40^b^	11.10^a^	12.40	0.51	0.008
Time_NV (in min)	90.40^a^	105.00^b^	80.60^a^	91.50	3.78	0.004
Time_NNV (in min)	61.40	88.20	63.50	71.30	4.80	0.94
Avg_NNV (in min/visit)	4.94	5.90	5.35	5.44	0.30	0.79
Avg_NV (in min/visit)	37.70^a^	34.00^a^	33.50^b^	34.00	1.15	0.08
Rank_cat	10.60	9.00	9.60	9.60	0.21	0.99
Features from automatic video analysis (used for decision tree)						
Side_lying (%)	51.00	50.10	50.20	50.40	0.21	0.29
Ventral_lying (%)	21.30^a^	22.00^b^	21.20^a^	21.50	0.13	0.06
Standing (%)	19.20	18.60	20.40	19.50	0.12	0.49
Sitting (%)	1.13	0.93	0.93	0.98	0.02	0.10
Eating (%)	3.20^a^	3.93^b^	2.97^a^	3.31	0.04	0.03
Drinking (%)	0.58	0.66	0.60	0.61	0.01	0.23

**Figure 2:**
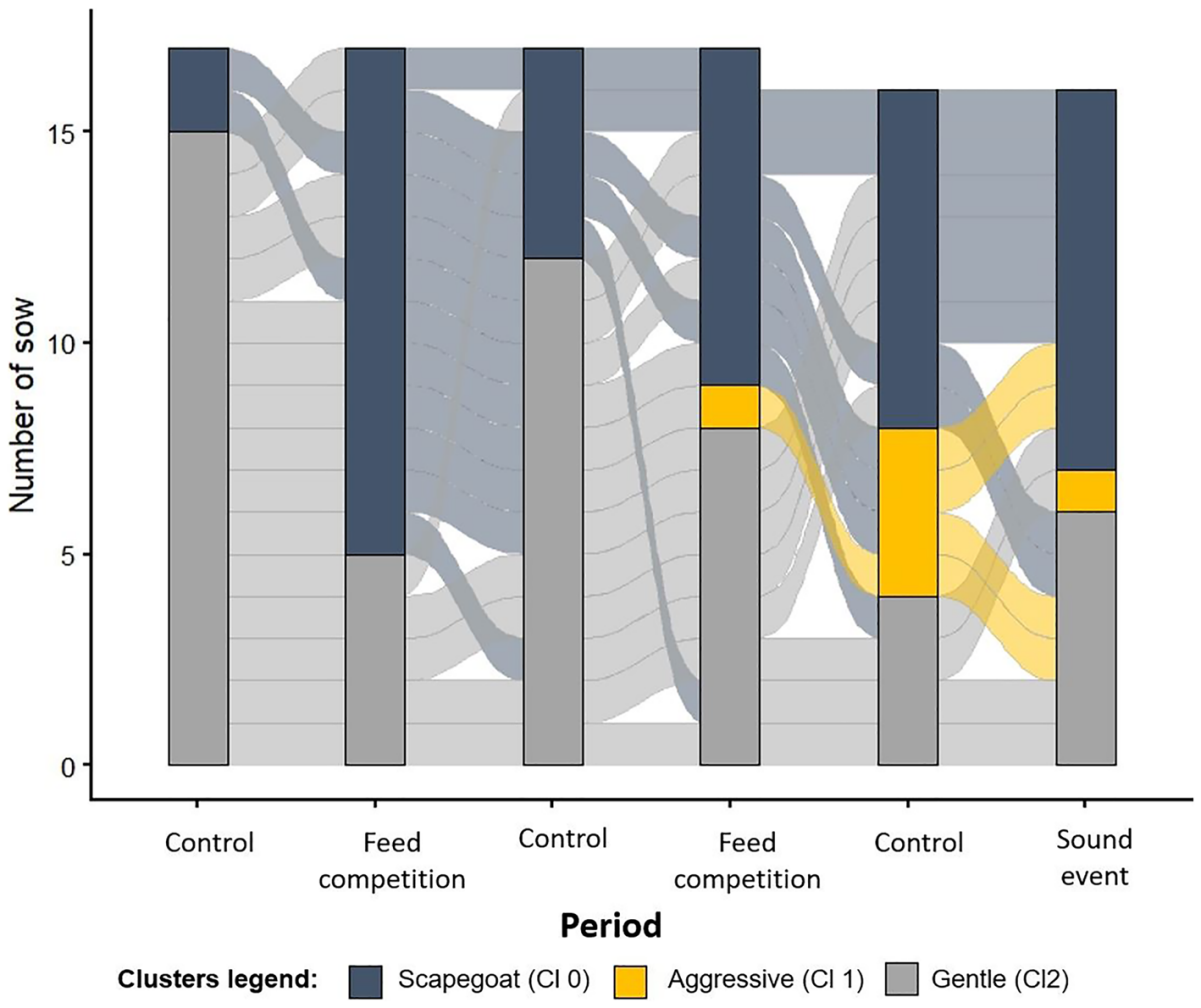
Evolution of the sows from group A between the three clusters during the six periods followed. One line between periods corresponds to one sow evolution.

The evolution of sows’ cluster attribution in group A during the 6 periods showed differences between periods and sows (Fig. 2). For example, one sow (the bottom line) was always classified as a ‘gentle’, while a majority of sows changed between ‘gentle’ and ‘scapegoat’ clusters due to the alternation of control (1, 3 and 5) and event (2, 4, and 6) periods. The evolution graph also showed that sows did not stay ‘aggressive’ for more than two periods.

### Interpretation of the clusters and link with welfare

**Figure 3:**
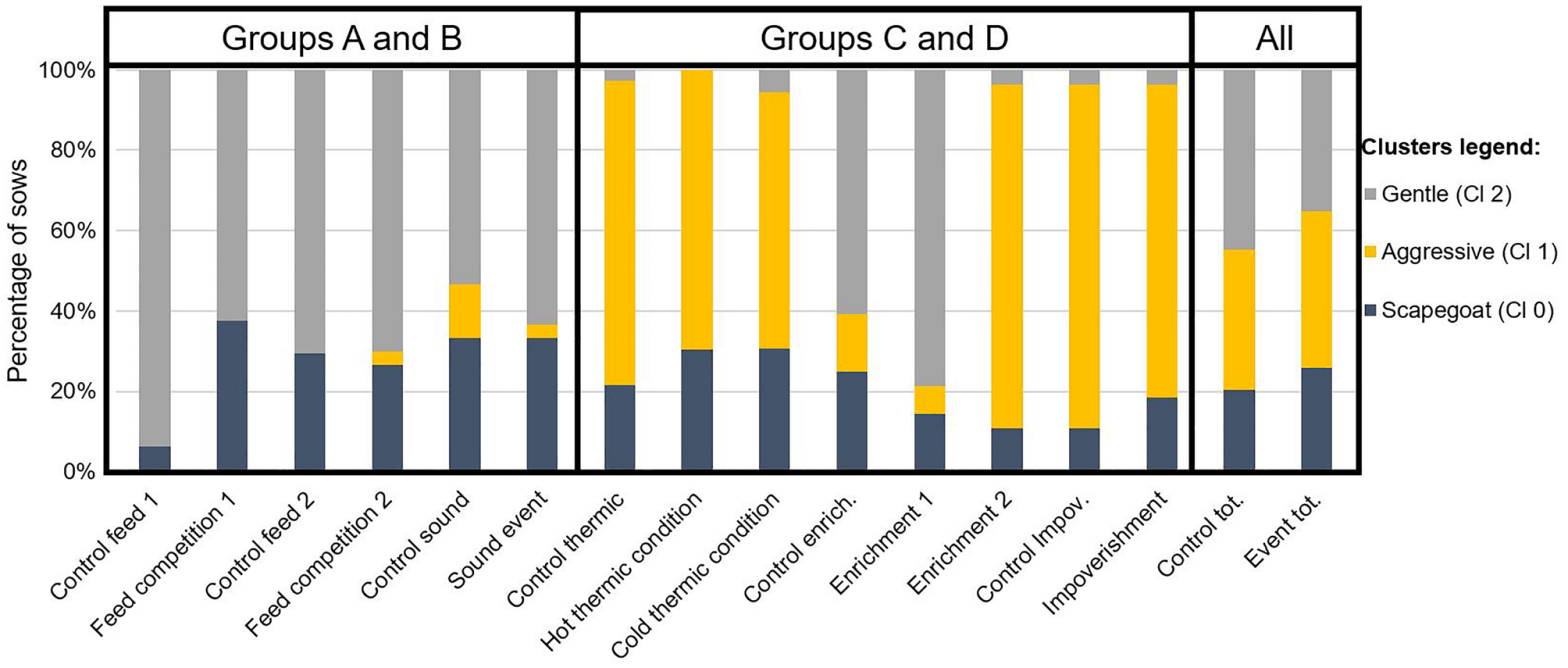
Proportions of sows in the three clusters (gentle, aggressive, scapegoat) for each week (control or events).

There was a significant dependence between the weeks (control vs. event) and the clusters (P < 0.01). During the event weeks, the proportion of ‘gentle’ sows tended to be lower compared to control weeks (53% vs. 35%, P = 0.07), while the proportion of ‘scapegoats’ (22% vs. 26%, P > 0.10) and ‘aggressive’ sows tended to increase (25% vs. 39%, P = 0.07, Fig. 3).

Groups A and B had a higher proportion of ‘gentle’ sows than groups C and D (96% and 50%, respectively, P < 0.001) and a lower proportion of ‘aggressive’ sows (49% and 75%, respectively, P < 0.001). While there were no differences between C and D (P > 0.10), there were significant differences between A and B (P < 0.01). Compared to group B, group A had more ‘scapegoats’ (4% vs. 43%) and more ‘aggressive’ (0% vs. 6%) and fewer ‘gentle’ (98% vs. 50%) sows.

There was a significant dependence between the type of event (feed competition, sound event, thermic variation, enrichment and impoverishment) and the clusters (P = 0.02, Fig. 3). A higher proportion of ‘gentle’ sows were found during the feed competition and sound events (66% and 63%) than during the other events, heat, cold and enrichment/impoverishment events (P = 0.06). A higher proportion of ‘aggressive’ sows was found during the thermic events and impoverishment (67% and 78%, P = 0.01) than during the other events (average 4%).

There were no effects of body weight, backfat thickness, age of the sow or hierarchical rank (P > 0.10, Table 2) on the clusters. The health of the sows tended to be different between the clusters. Indeed, the unhealthy sows (bursitis, tail biting, vulvar lesions, lameness) were more aggressive than healthy sows (28 vs. 12%, respectively, P = 0.05). This difference was particularly linked to the fact that more unhealthy sows suffered from tail biting and vulvar lesions. Therefore, the behavioral clusters could be representative of the different states of welfare and be used for the automatic classification task.

### Automatic classification of welfare status

The decision tree classification results with the feeder and the automatic video analysis data had accuracies of 80% and 72% (for the training and testing datasets respectively, using the labels from the clustering step) and F1-scores of 0.80 and 0.72. The performance results by clusters showed better results to predict ‘gentle’ and ‘aggressive’ sows (F1-score = 0.86 and 0.80, respectively) than ‘scapegoat’ (F1-score = 0.70). The decision tree with the feeder data only had accuracies of 67% and 64% for the training and testing datasets, respectively, and F1-scores of 0.61 and 0.56. The performances to predict ‘scapegoat’ were better with feeder data only than with feeder and automatic video analysis data together, while this combination of measurements gave higher performances for ‘aggressive’ and ‘gentle’ sows (Table 3).

**Table 3:** Performance metrics (precision, recall, F1-score) depending on the clusters and the data used in the decision tree. Higher performances values are in bold.

Clusters	Feeder + automatic video analysis			Feeder only		
	Precision	Recall	F1-score	Precision	Recall	F1-score
0 (scapegoat)	0.60	0.85	0.70	**0.69**	**0.97**	**0.80**
1 (aggressive)	**0.83**	**0.77**	**0.80**	0.58	0.29	0.39
2 (gentle)	**0.97**	**0.78**	**0.86**	0.60	0.10	0.18

**Figure 4:**
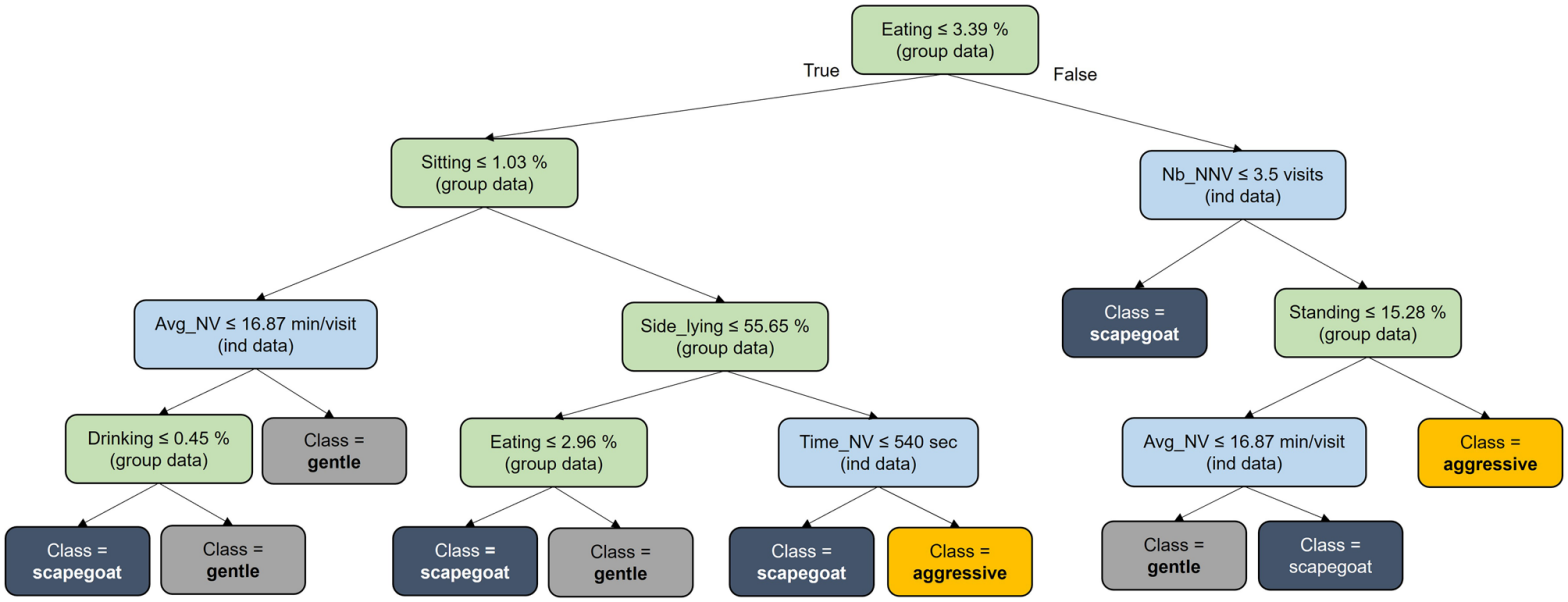
Decision tree to predict clusters with the feeder (individual data in blue) and automatic video analysis data (group data in green).

The decision tree (Fig. 4) showed the paths in the tree, through the various possible values of group and individual variables, to reach the class of the sow. For instance, a sow was classified as ‘aggressive’ by starting from the root node through the right branch of the tree to the leaf node “aggressive”. It means that if the sows of the group spend more than 3.39% of their daily time eating, and if the sow did more than 3.5 non-nutritive visits per day to the feeder, and if the sows of the group spent more than 15.28% of their daily time standing, then it is classified as “aggressive”.

## Discussion

The results showed that estimating the welfare status of gestating sows during a specific period may soon be possible. Using clustering in conjunction with classification on automatically recorded on-farm data opens the door to innovative research opportunities^37^. The behavior features used to build clustering (level of physical activity and social interactions) were found in the literature to be linked to some components of welfare^13,21–24,38^. The principal concern regarding this welfare evaluation was the variability in threshold definitions, which can differ based on farm conditions or groups of sows. An advantage of data mining methods (such as clustering) is that they can be readily applied to farm data owing to their unsupervised learning nature, which does not require any prior knowledge or data annotation. However, one of the drawbacks of data mining for cluster interpretation is that the clusters generated may not always have straightforward and easily interpretable meanings. To validate this new and upgradeable first approach, its application on additional datasets will be necessary to improve the studied link between welfare and behavior. To establish a more robust method, it should be testing with respect to other identified welfare indicators in the literature, such as stereotypies (abnormal behaviors linked to boredom)^21^, which can also be measured through video analysis.

The three clusters identified in this study demonstrated a correlation with health status, particularly for ‘aggressive’ behavior. Animals’ health status was considered a part of welfare status^39^, and behavioral changes could be potential signs of illness^40^. The characteristics of the sow did not significantly influence the clustering: as a result, a sow could belong to one of the three behavioral clusters depending on the studied period. This is in accordance with one welfare definition^41^ and showed that the clustering method may be an estimation of some components of the welfare status of an individual at a given time. However, this outcome is unexpected, considering that numerous studies have demonstrated the impact of parity on physical activity or agonistic interactions, which are variables used for the clustering ^42–44^. However, the experimental design does not reveal any causal links but only potential correlations.

With these different proportions of sows in clusters, discrimination may be achievable not only between controls and events but also among different types of events. The effect of the event on the sow’s behavior was reported in other studies. For example, the increased competition for feed resources could increase agonistic social relations and the level of physical activity, inducing degradation of welfare status^45^. In contrast, the enrichment of the pen decreases agonistic social relations and improves welfare status^11^. If these results also suggest this approach could be a method to estimate the welfare status of farms, validation on farms with varying environmental conditions must be conducted to rule out any bias in this experimental design.

The group effect on individual behaviors is also of major interest. Group behavior could have an impact on the behavior of all animals and may depend on the composition or size of the group^42^. The difference in the proportion of ‘aggressive’ sows between groups A and B vs. groups C and D, during event or control periods, may suggest an important effect of the group of sows on the individual welfare status. The effect of group may be explained to the difference in events induced between groups A and B vs. groups C and D.

Predictions made using a learned decision tree on combined feeder and automatic video analysis data yielded satisfactory results (with an accuracy of over 80%). The importance of feeding behavior and activities to determine health or welfare was also confirmed by Matthews et al.^46^. Due to the restricted feeding of sows, the number of nonnutritive visits and the time of eating (duration of nutritive visits) were key features to classify components of welfare. The introduction of group behavioral features (from automatic video analysis) in the decision tree model decreased the prediction performance of the ‘scapegoat’ compared to the feeder data only (F1 score = 0.60 vs. 0.80). One hypothesis could be that the ‘aggressive’ and ‘gentle’ classes are mainly linked to group behavior, and the ‘scapegoat’ class is mainly linked to individual features. This ‘scapegoat’ behavior was also not significantly linked to the type of week (control/event) or health status. It would be the most difficult class to characterize and to predict due to the lack of link with environmental conditions. On the decision tree, the importance of group behavior on individual behavior was also shown with the importance of group level features. Therefore, as pig is a social animal, group behavior may have an impact on the welfare of each sow^21^ and is required to take it into account to predict the individual welfare status.

## Conclusions

This study represents a significant step toward estimating the welfare status of gestating sows, particularly with the rapid advancements in the field of artificial intelligence within the realm of animal science. The approach of employing machine learning techniques, which combines an unsupervised method like clustering for data labeling with a supervised method for sensor data to learn an interpretable decision tree, yields meaningful results. However, further efforts are needed to fully interpret these clusters as “welfare estimators” and utilize them for distinguishing environmental conditions or health issues. In practice, this method could be integrated into a decision support system (DSS) to comprehensively monitor the living conditions of animals. Furthermore, the DSS can predict potential issues and alert farmers to welfare concerns, thus contributing to proactive and preventive animal welfare management.

## Data Availability

The data used in this study are available for public access and described in the data paper from Durand et al.^23^.
